# How resilient were family planning programs during the COVID-19 pandemic?  Evidence from 70 countries

**DOI:** 10.12688/gatesopenres.14856.1

**Published:** 2023-09-28

**Authors:** Karen Hardee, Rebecca Rosenberg, John Ross, Imelda Zosa-Feranil

**Affiliations:** 1 Hardee Associates, Arlington, VA, 22207, USA; 2 Avenir Health, Glastonbury, Connecticut, 06033, USA; 3 Independent demographic consultant, New Paltz, NY, 12561, USA

**Keywords:** Family planning, COVID-19, national composite index of family planning, NCIFP

## Abstract

**Background**: At the beginning of the COVID-19 pandemic fears of severe disruptions to family planning (FP) and access to services abounded. This paper uses a unique data source, a special Supplement added to the 2021 round of the National Composite Index for Family Planning (NCIFP), to assess in depth the resilience of FP programs in the face of the COVID-19 pandemic across 70 countries spanning six regions.

**Methods**: The 2021 NCIFP included 961 key informants who were asked questions to assess interference in the countries’ ability to achieve objectives, ability to maintain commitment to FP, and availability of information and services. Open ended responses added context.

**Results**: All programs were affected; the magnitude of effects varies by region and country. While the average resilience score, at 47 out of 100, implies middling levels of resilience, further analysis showed that despite interference in many components of programming, with some exceptions, the COVID-19 pandemic generally did not diminish government commitment to FP and programs remained resilient in providing access to services. Common themes mentioned by 178 respondents (18.5% of respondents) included: fear of infection; disruption of services / difficulty with lockdown and travel restrictions; staff / facilities diverted to COVID-19; access to reproductive health services and contraceptive methods affected; shifts in services / outreach; interference with logistics & supplies, training & supervision, and M&E; lack of attention to FP/sexual reproductive health; financing reduced or diverted; and effects on existing partnerships. A strong enabling environment for FP, which the NCIFP is designed to measure, was positively correlated with continued government commitment and access to contraceptive methods during COVID-19.

**Conclusion**: These findings are instructive for programming: it will face challenges and ‘interference’ when unanticipated shocks like COVID-19 occur, with strong FP programs best prepared to exhibit resilience.

## Background

In 2020, when the global COVID-19 pandemic was unfolding and was taking a toll on health systems, communities and individuals, questions arose about how family planning programs and contraceptive services would fare in the face of COVID-19 ( Brunie *et al.*, 2022; UNFPA, 2021; GEH *et al.*, 2021). One estimate suggested that 15 million unintended pregnancies over a year could result from a 10 percent reduction in use of contraception in lower- and middle-income countries ( Riley *et al.*, 2020), raising dire warnings of strong negative consequences associated with anticipated disruption of access to contraceptives. WHO conducted pulse surveys on disruptions to essential sexual reproductive maternal neonatal children and adolescent health services, once in mid-2020 and twice in 2021. These pulse surveys show widespread disruptions to family planning services early in the pandemic, diminishing over time ( WHO, 2021b). In the first pulse survey in May-July, 2020, 68 percent of the 102 states that responded noted disruptions in family planning and contraceptive services, compared to 44 percent of the 104 countries responding in January-March 2021 ( WHO, 2020; WHO, 2021a). By November-December 2021, 35 percent of the countries responding noted disruptions ( WHO, 2021b). A multi-country study of COVID-19 and resilience of health systems found large declines in family planning services in two countries (Chile and Mexico), a small decline in one country (Nepal) and no decline in four countries (Haiti, Lao PDR, Ethiopia and Ghana) ( Arsenault *et al.*, 2022). Country respondents reported that most effects to the health systems, including related to family planning, had been addressed by the end of 2020, with more lasting effects in Chile and Mexico.

Evidence from country-level studies suggest mixed findings on effects of the COVID-19 pandemic on contraceptive use ( Bietsch *et al.*, 2022; Brunie *et al.*, 2022; Karp *et al.*, 2021; Wood *et al.*, 2021). A voice response survey to gain insight into women’s contraceptive access and use in Malawi, Nepal, Niger, and Uganda one year into the pandemic, found less reported use of contraception than before the pandemic in three of the four countries (Niger was the exception) ( Brunie *et al.*, 2022). The sample in the Brunie *et al.* study was primarily younger women ages 18 to 24. Temporary service closures, product shortages, and fear of COVID-19 infections were reported to affect access and use. Using multiple rounds of Performance Monitoring and Action (PMA) data from Kenya, Burkina Faso, Lagos State Nigeria and Kinshasa, DRC, Wood *et al.* (2021) reported some increases in women’s need for contraception. However, results also showed continued increases in contraceptive use. Assessing contraceptive use in 15 countries using service statistics data from 2019 and 2020, Bietsch *et al.* (2022) found that contraceptive use was higher in 12 of the countries in 2020 than in 2019.

Most studies that have asked about family planning program disruptions have not delved deeply into what aspects of the program have been disrupted (e.g., the WHO pulse surveys ask only one question), and the more in-depth evidence that is available covers a handful of the countries around the world, or is summarized without specific country information ( Global Health Supply Chain Program, n.d.). This paper uses a unique data source, a special Supplement added to the 2021 round of the National Composite Index for Family Planning (NCIFP), to assess in depth the resilience of family planning programs in the face of the COVID-19 pandemic across 70 countries spanning six regions: Francophone sub-Saharan Africa; Anglophone sub-Saharan Africa; Asia; Latin America and the Caribbean; Middle East and North Africa, and Eastern Europe and Central Asia.

In this paper, we first explain the NCIFP then present findings from the Supplement added to assess the effects of COVID-19 and thus the resilience of the family planning program during COVID-19, set in the context of the findings of the general NCIFP in 2021.

## Methods

### The National Composite Index of Family Planning

Building on the Family Planning Program Effort Score measured since the 1970s ( Kuang & Brodsky, 2016; Lapham & Mauldin, 1984; Mauldin & Ross, 1991; Ross & Stover, 2001), the NCIFP was developed after 2012 to support FP2020’s efforts to improve the enabling policy and program environment for family planning, by examining the levels and types of effort for a range of family planning policy and programmatic indicators, including indicators to measure rights-based programming.

The NCIFP includes 41 items related to five dimensions of family planning programs, namely, Strategy, Data, Quality, Equity and Accountability, with scores summing to a total possible score of 100 ^1^ ( Box 1). A ‘COVID-19 Supplement’ was added to the 2021 round ( Rosenberg *et al.,* 2022). The first two rounds of the NCIFP were conducted in 2014 and 2017 ( Rosenberg, 2020). For more information about development of the NCIFP, see Weinberger & Ross (2016).

Box 1. **The 5 NCIFP Dimensions + 2021 COVID-19 Supplement**

   ■ **Strategy:** Includes questions on topics such as what plans are in place, whether they include important elements (e.g. quantified objectives), government support for family planning, etc.
   ■ **Data:** Focuses on both data collection (service statistics, monitoring sub-groups, etc.), and data use to inform decisions.
   ■ **Quality:** Measures whether services meet WHO standards, whether quality of care indicators are monitored, whether there are structures are in place to support quality services.
   ■ **Equity:** Focuses on policies and programs related to discrimination, efforts to reach under-served groups, and wide-spread access to contraceptive methods.
   ■ **Accountability**: Focuses on monitoring and addressing issues related to informed choice, lack of coercion and absence of denial of services.
   ■ **COVID-19 Supplement (questions included in the 2021 round):** Asks to what extent COVID-19 affected various components of the family planning program.

The 2021 round of the NCIFP includes results from 70 countries in six regions, a drop from 84 countries in the 2017 round. The lower number of countries in 2021 stemmed from challenges associated with the COVID-19 pandemic and the war in Ukraine. The regional representativeness of the sample for 2021 is 64 percent of countries for both Anglophone and Francophone sub-Saharan Africa regions (SSAF-A and SSAF-F), 52 percent of countries for Asia, 18 percent of countries for Latin America and the Caribbean (LAC), 21 percent of countries for the Middle East and North Africa (MENA), and 53 percent of countries for Eastern Europe and Central Asia (EECA). The total number of respondents in 2021 was 961.

The NCIFP is administered through a key informant approach, with the key informants comprising 10–15 respondents in each country who know the family planning program, from the public sector and private sectors; from civil society and nongovernmental organizations; from academic/research organizations; and from development and implementation partners. The approach uses a rating system based on opinion of “the extent to which” with 1–10 as responses (1 = non-existent; 10 = extremely strong effort) for each item in the NCIFP. The 2021 round was administered in eight languages online using Google Form, with an offline option available. The questionnaire included informed consent for respondents.

### COVID-19-specific questions added to the 2021 NCIFP as a Supplement

The Supplemental questions on COVID-19 came at the end of the regular NCIFP questionnaire and respondents were told that this final set of questions was unique to the 2021 NCIFP and was meant to capture the resiliency of the health system. The questions on COVID-19, shown in Box 2, covered four main aspects of family planning program resiliency: 1) the extent to which COVID-19 interfered with the country’s ability to achieve its objectives related to seven issues (shown in Box 2), with space to add additional issues; 2) the extent to which the government maintained commitment to family planning; 3) the extent to which the family planning program was able to maintain availability of contraceptive information and services; and 4) extent to which clients were able to access contraceptive counseling and methods during lockdowns associated with COVID-19. Taken together, these results give an indication of the resiliency of family planning programs in the face of the COVID-19 pandemic from the perspective of stakeholders who know the programs well.

Box 2. **COVID-19 Supplement Items in the 2021 NCIFP to Measure Resilience of the Family Planning Program During COVID-19**

- Extent to which COVID-19 interfered with the country's ability to reach its family planning objectives. (1 = not at all; 10 = extremely interfered)
  • Financing for family planning
  • Advocacy or community mobilization efforts
  • Supply of contraceptives, including transport and logistics systems
  • Recording and reporting of services (routine data)
  • Restrictions to movement/transport that interfered with the population’s access to short-term FP methods (STM)
  • Restrictions to movement/transport that interfered with the population’s access to long-term and permanent family planning methods (LAPM)
  • Other (please specify)
- Extent to which the government maintained its commitment to family planning during COVID-19. (1 = not at all; 10 = maintained commitment)
- Extent to which the family planning program was able to maintain availability of contraceptive information and services, including contraceptive methods during COVID-19. (1 = not at all; 10 = availability maintained)
- Extent to which clients were able to access contraceptive counseling and methods during lockdowns associated with COVID-19. (1 = no access; 10 = easy access)

### Analysis of the NCIFP

Data from the google forms questionnaires were exported to Excel for analysis. The authors, led by RR, calculated scores for each item by averaging across individual items in each dimension of the NCIFP and across the COVID-19 Supplement questions. Total scores are an average across all 41 items in the NCIFP. Dimension scores are an average of the individual items in each dimension. Country scores were generated by taking the average for all respondents within that country and converting to a score from 1-100. Regional scores are the average of all country scores within that region. Scoring on responses to COVID-19 questions about ‘interference’ was reversed for analysis for consistency with scoring of the five dimensions of the NCIFP. Thus, a high score indicates little interference to the program from COVID-19 and thus higher resilience of the program and a low score indicates a great deal of interference and lower resilience.

Analysis also included correlations between the NCIFP total score (an average of the five dimensions (excluding the COVID-19 Supplement) and three COVID-19 Supplement indicators: 1) the total Supplement score; 2) the Supplement item on whether government commitment was maintained; and 3) the Supplement item on whether access to contraception was maintained.

Respondents had the opportunity to provide a short answer to ‘COVID Other’ following the questions about COVID-19 interference. The authors, led by KH, analyzed the responses to this open-ended question to highlight common and unique themes across the regions. We started with a list of all of the open-ended responses, grouped by country and region. Starting with the themes represented by the items in the COVID-19 Supplement, we let the comments ‘speak for themselves’ and represent the voices of the respondents in terms of themes that emerged.

### Ethical approval and consent

Since the first round in 2014, the NCIFP has been conducted within a monitoring and evaluation framework focused on family planning programs, rather than under a research protocol. Still, written informed consent was obtained to take part in the NCIFP and all data has been anonymized.

## Findings

### Regional variation in family planning program resilience during COVID-19

**Figure 1.**
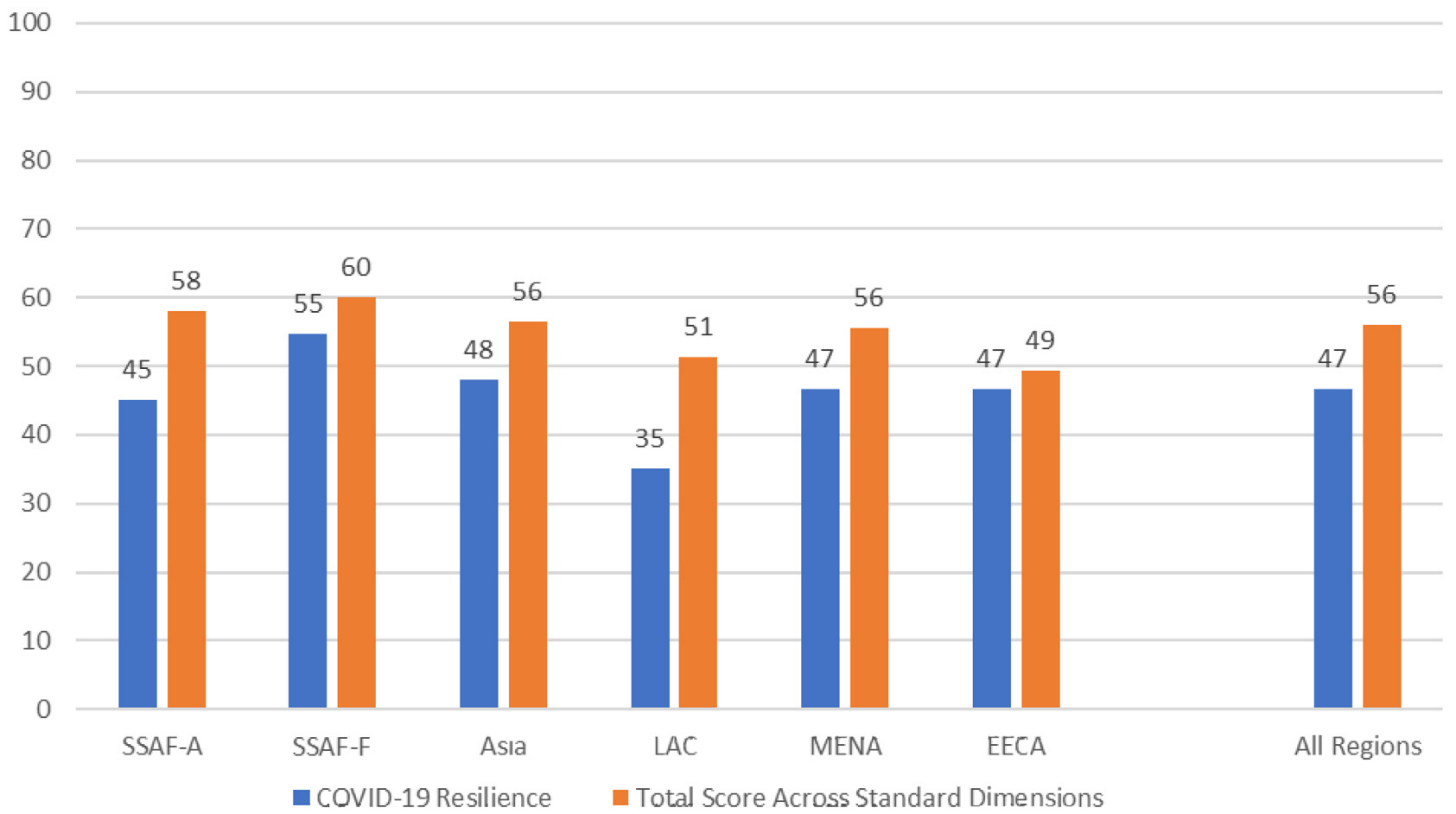
Resilience of the FP Program during COVID-19 and NCIFP Score, by Region.

Regions show variations in the resilience of their family planning programs during the COVID-19 pandemic ( Figure 1). Keeping in mind that a high score indicates higher resilience and a low score indicates lower reliance, the average score across all 70 countries was 47 out of 100. SSAF-F had the highest score (55), indicating that that region’s family planning programs were affected by COVID-19, but still may have been most resilient during the pandemic. LAC has the lowest overall score (35), meaning that COVID-19 was considered to affect family planning programs to a greater extent in that region. Figure 1 also shows that respondents gave higher scores overall for the five standard dimensions of the NCIFP in 2021 (described in Box 1) compared to their assessments of the resilience of the program in the face of COVID-19.

No region or country was immune to effects of COVID-19 on the family planning program. Regions showed variation in the resilience of their programs, ranging from a high of 55 in Francophone sub-Saharan Africa (out of 100 indicating fully resilient) to a low of 35 in Latin America and the Caribbean, with an average across the six regions of 47.

**Figure 2.**
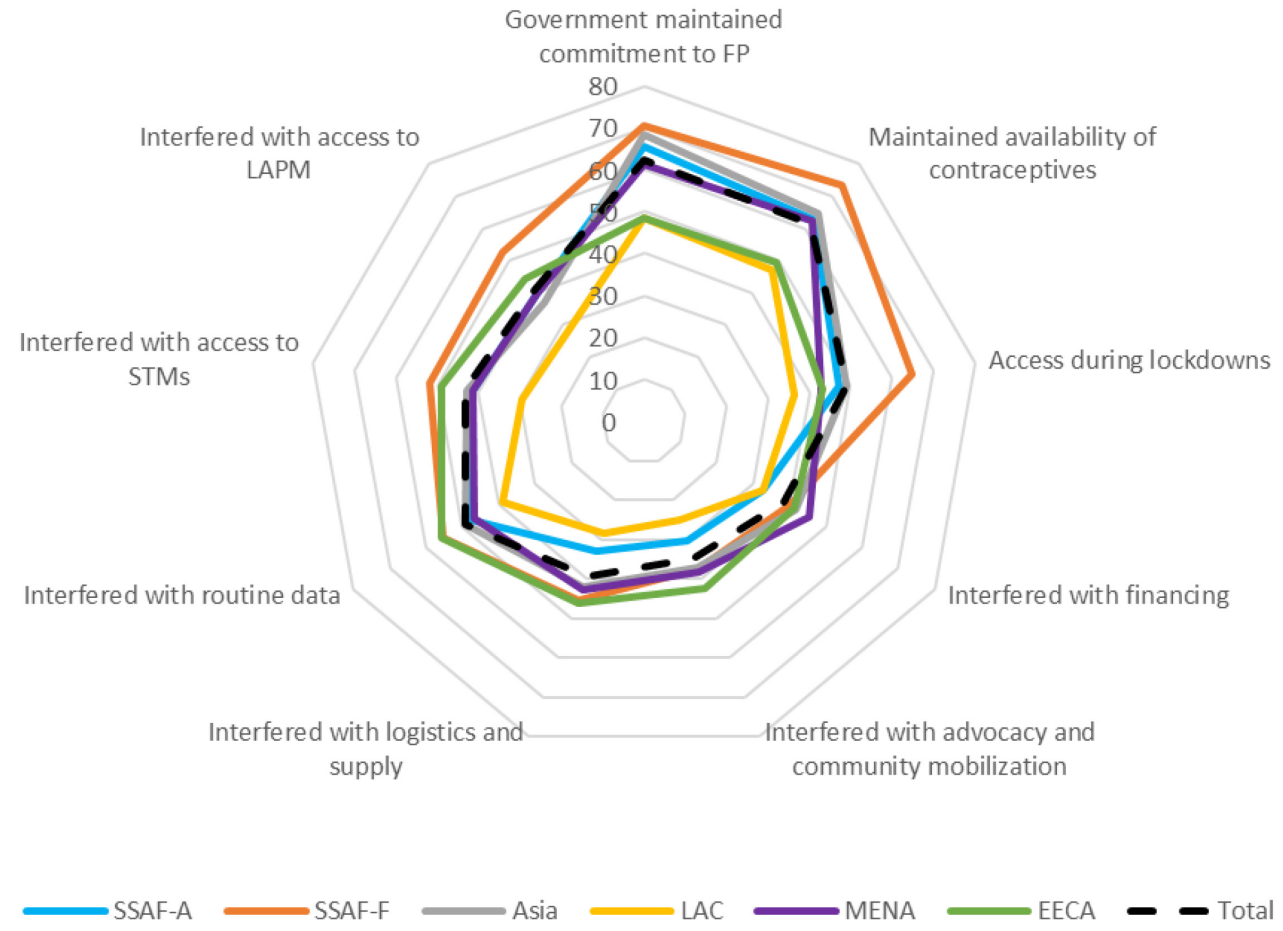
Components of resilience, by Region. Note: The wording for each component of resilience in Figure 2 is found in Box 2.

Turning to the components of resilience of the family planning program to COVID-19 ( Box 2 and Figure 2), respondents in four of the six regions were positive about their governments’ continued commitment to family planning and ability to maintain access to contraceptives in the face of COVID-19. LAC (yellow line) and EECA (green line) were the exceptions, with less positive views on government commitment and access to contraceptives during COVID-19 ( Figure 2). Regarding interference, across the regions, COVID-19 was considered to have the most effect on advocacy and community mobilization efforts and on supply and logistics. Restrictions to movement and/or transport were considered to have had more effect on access to long acting and permanent methods (LAPM) than on access to short term methods (STM) across all regions except SSAF-F (orange line). SSAF-F had equal levels of interference to access to both types of methods related to restrictions to movement and/or transport.

Respondents in four of six regions were positive about government commitment to family planning and access to contraceptives during COVID-19 although all regions reported interference with logistics and supply and, relatedly, access to both types of methods due to restrictions on movement and/or transportation.

### Country variation in family planning program resilience during COVID-19

**Figure 3.**
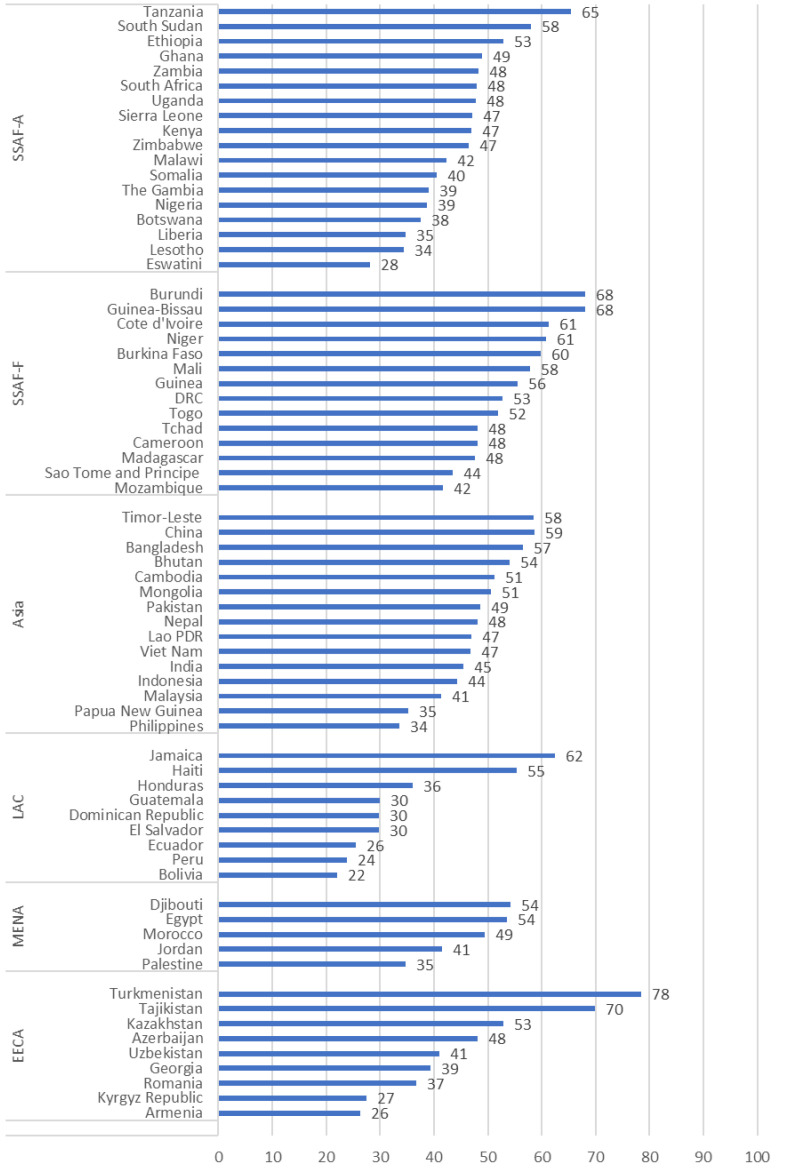
Resilience of the FP Program During COVID-19, for Countries Grouped Within Regions.

Country scores for resilience vary widely, with Turkmenistan reporting the highest resilience (78) and Bolivia the lowest (22) ( Figure 3). Within regions, there is considerable variation in the country scores for resilience. The largest difference is 52 points in EECA, from the high of 78 for Turkmenistan and a low of 26 for Armenia. The smallest difference is 19 points in MENA (from a high of 54 in Djibouti to a low of 35 in Palestine. Table 1 provides the component scores for the items in the COVID-19 Supplement for each country, grouped within regions. The scores across countries reinforce that for most countries, with some exceptions, while the government maintained commitment to family planning, the programs faced interference. Among the 70 countries, around half (36) had similar scores related to the government maintaining commitment to family planning and the country maintaining availability of contraceptives (scores for those two items were within 5 points of each other in those 36 countries). In eight countries, the scores for those items differed by 10 or more points.

**Table 1.** Resilience of the FP Program During COVID-19, Component Scores by Country within Region.

	COVID-19 interfered with…						Government maintained commitment to FP	Maintained availability of contraceptives	Access during lockdowns	Average score
Region and Country	financing	advocacy	logistics and supply	routine data	access to STMs	access to LAPM				
SSAF-F	40	38	46	55	52	53	71	73	65	55
Mozambique	26	24	24	47	29	31	67	64	61	42
Sao Tome and Principe	16	17	29	31	33	36	79	86	64	44
Madagascar	32	29	38	43	44	46	50	76	71	48
Cameroon	36	30	38	56	46	50	61	63	53	48
Tchad	42	40	44	44	53	56	47	58	48	48
Togo	40	35	39	55	48	45	73	75	58	52
DRC	46	51	44	61	52	54	56	65	45	53
Guinea	44	44	46	58	51	50	74	69	64	56
Mali	36	26	52	54	58	55	82	81	76	58
Burkina Faso	48	44	50	59	57	53	79	79	70	60
Niger	33	37	59	64	65	66	78	77	69	61
Cote d'Ivoire	51	42	50	63	63	63	78	71	70	61
Guinea-Bissau	56	52	67	69	61	62	81	84	79	68
Burundi	58	51	56	70	67	67	84	79	79	68
SSAF-A	33	30	33	47	41	40	65	63	47	45
Eswatini	12	16	22	39	15	21	60	47	21	28
Lesotho	21	30	30	36	35	32	42	49	32	34
Liberia	21	22	24	31	27	29	61	57	41	35
Botswana	24	22	20	37	28	30	76	58	43	38
Nigeria	31	29	32	42	36	33	50	55	41	39
The Gambia	20	22	26	37	27	29	67	74	49	39
Somalia	35	32	27	50	57	54	38	41	29	40
Malawi	28	32	27	51	40	36	60	61	45	42
Zimbabwe	35	31	31	43	29	25	86	81	57	47
Kenya *	43	43	43	43	43	43	62	60	41	47
Sierra Leone	34	36	36	49	41	42	69	71	45	47
Uganda	41	32	33	55	44	41	75	63	45	48
South Africa	43	33	42	46	44	36	66	69	52	48
Zambia	46	32	48	46	32	45	69	54	61	48
Ghana	32	33	43	61	50	49	67	60	46	49
Ethiopia	32	31	33	54	52	43	83	82	66	53
South Sudan	48	33	43	57	74	66	67	74	60	58
Tanzania	52	46	44	72	73	67	80	79	75	65
Asia	42	37	42	49	43	37	68	65	49	48
Philippines	19	20	21	22	22	8	71	66	53	34
Papua New Guinea	27	25	31	52	31	31	38	44	38	35
Malaysia	36	29	42	51	33	34	51	54	43	41
Indonesia	31	30	29	34	31	26	79	74	66	44
India	58	46	46	44	42	35	57	45	35	45
Viet Nam	23	28	36	39	42	38	78	73	65	47
Lao PDR	34	35	30	33	48	47	73	66	56	47
Nepal	43	33	39	54	44	31	69	68	52	48
Pakistan	42	52	36	55	44	34	73	57	44	49
Mongolia	49	57	34	58	47	50	53	59	48	51
Cambodia	41	36	52	67	52	43	69	68	35	51
Bhutan	49	27	50	56	44	37	87	79	57	54
Bangladesh	56	43	69	59	52	43	77	66	44	57
China	59	46	57	65	59	47	72	75	48	59
Timor-Leste	60	50	59	50	52	47	81	75	52	58
LAC	33	25	28	39	30	28	48	47	37	35
Bolivia	16	14	15	37	16	18	27	31	25	22
Peru	22	10	16	17	18	18	49	40	24	24
Ecuador	18	19	23	24	25	21	36	37	27	26
El Salvador	29	31	30	34	27	25	29	35	28	30
Dominican Republic	32	12	18	26	17	16	64	48	35	30
Guatemala	46	19	21	41	10	11	41	44	38	30
Honduras	28	22	24	45	30	28	52	54	40	36
Haiti	48	47	55	63	63	59	55	61	47	55
Jamaica	53	53	54	63	60	56	84	76	64	62
MENA	46	38	43	47	41	40	61	62	43	47
Palestine	35	37	29	31	26	26	47	45	37	35
Jordan	39	34	46	54	40	27	47	57	31	41
Morocco	45	39	50	41	40	42	71	73	44	49
Egypt	62	39	41	58	43	46	72	70	52	54
Djibouti	48	42	47	49	59	57	70	66	50	54
EECA	42	42	46	56	49	44	49	49	43	47
Armenia	13	19	16	29	38	38	26	30	28	26
Kyrgyz Republic	18	21	24	28	20	20	46	37	33	27
Romania	42	30	51	62	39	29	18	32	27	37
Georgia	38	42	49	54	42	39	27	36	29	39
Uzbekistan	35	37	33	51	42	36	47	50	37	41
Azerbaijan	38	51	40	47	53	52	56	47	47	48
Kazakhstan	33	36	60	57	51	49	66	65	60	53
Tajikistan	68	67	64	79	76	63	73	73	66	70
Turkmenistan	87	80	77	94	81	73	78	75	62	78

*Note that for Kenya, respondents were asked if COVID-19 interfered with the family planning program but not about how specifically it interfered; thus the same score is given for the six components related to interference.

Country scores for resilience vary widely, with Turkmenistan reporting the highest resilience (78 out of 100) and Bolivia the lowest (22). Even within regions, countries show considerable variation in their resilience score, and in the components of resilience that affect each country.

### Correlations between Total NCIFP Scores and COVID-19 Supplement Scores

**Figure 4.**
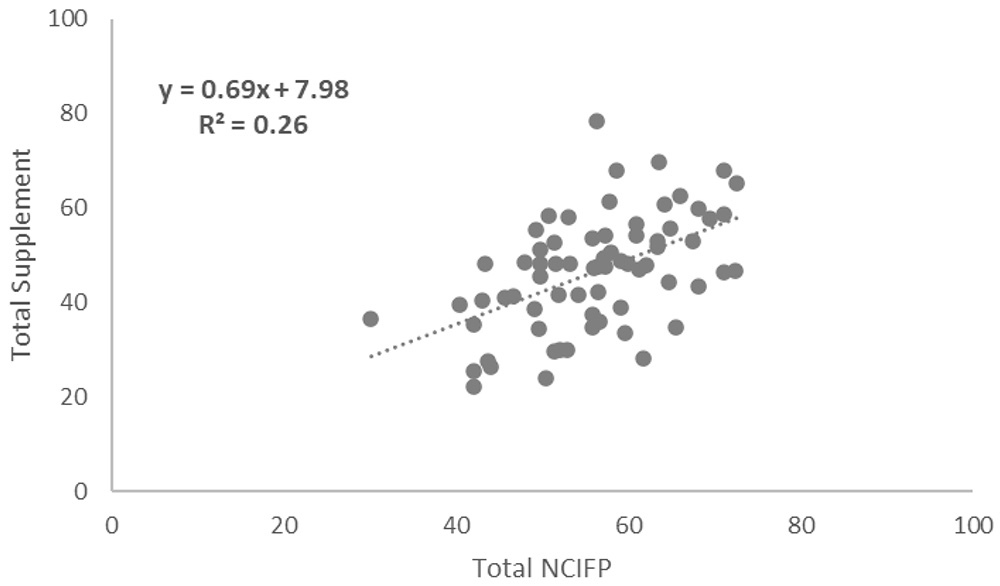
Correlation: Total NCIFP and Total COVID-19 Supplement.

**Figure 5.**
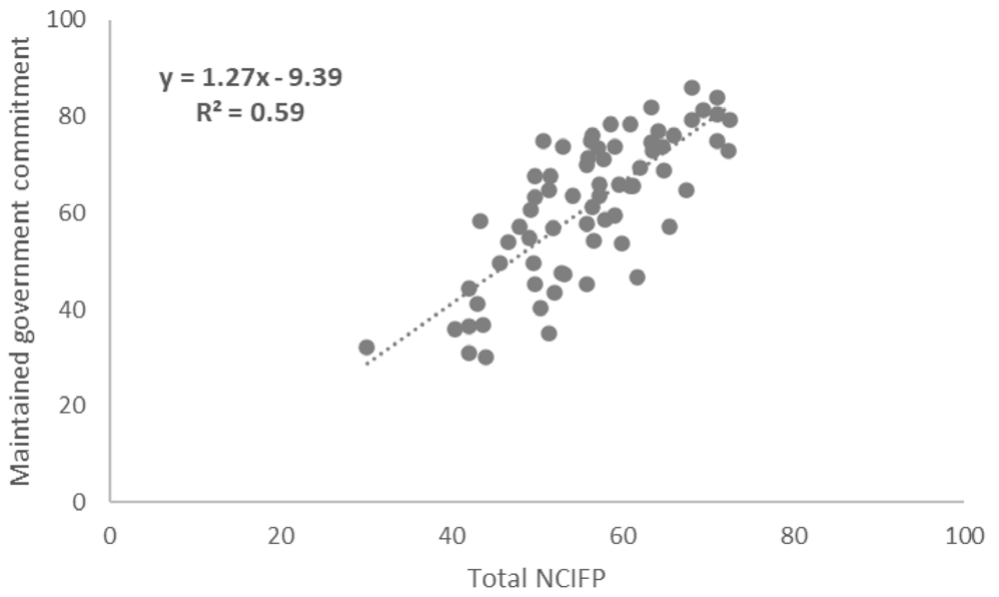
Correlation: Total NCIFP and Maintained Government Commitment During COVID-19.

**Figure 6.**
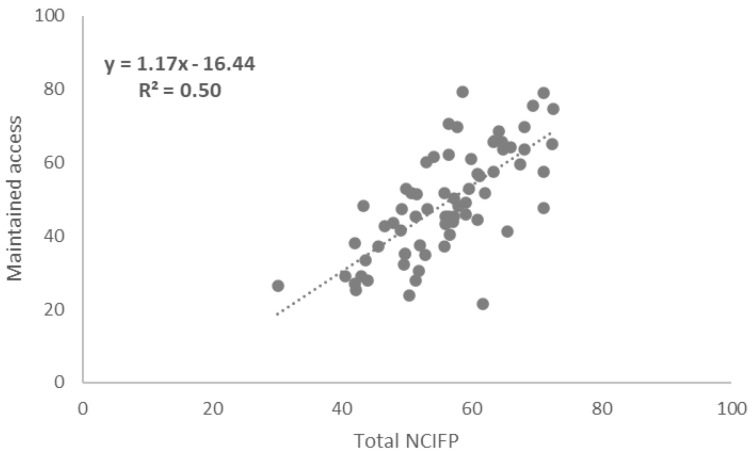
Correlation: Total NCIFP and Maintained Access During COVID-19

To assess links between the strength of the overall enabling environment for family planning in programs and resilience to COVID-19, we measured correlations between total scores on the five dimensions of the NCIFP (Strategy, Data, Quality, Accountability and Equity) and the Total COVID-19 Supplement score. The connection was positive with a correlation coefficient (R ^2^) of 0.26 ( Figure 4). The correlation between the total NCIFP Score and the Supplement item on whether government commitment was maintained was R ^2^= 0.59 ( Figure 5) compared to R ^2^= 0.50 for the Supplement item on whether access was maintained ( Figure 6). The relationships for all three were strong, implying that the stronger the enabling environment for family planning, as measured through the NCIFP, the more likely the family planning program was able to maintain commitment to family planning during COVID-19, and to maintain access to contraception.

Family planning programs with a strong enabling environment, as measured by the NCIFP, were more likely than those with weaker enabling environments to exhibit continued government commitment and access to contraceptive methods during COVID-19.

### Self-reported challenges related to the effects of COVID-19

Respondents were given the opportunity to provide open-ended responses about the effects of COVID-19 on family planning programming. Comments from 178 respondents from 63 of the 70 countries, representing around 18.5% of all respondents, provide a narrative snapshot and reinforcement of the challenges that family planning programs faced in the context of COVID-19 across the regions ( Table 2). Comments can be grouped into seven themes. Fear of infection was mentioned in 5 of 6 regions, disruption of services / difficulty with lockdown and travel restrictions was mentioned in all six regions and staff / facilities diverted to COVID-19 was mentioned in 4 of 6 regions. Five of six regions mentioned: access to reproductive health services and contraceptive methods affected; shifts in services / outreach affected; interfered with logistics & supplies, training & supervision, and M&E; and lack of attention to FP/SRH, financing reduced or diverted, and partnerships affected.

**Table 2.** Summary of comments, by Region *.

Theme	SSA-F	SSA-A	Asia	LAC	MENA	EECA
**Fear of infection**	Fear of going to health care settings (7)	Affected access to FP, most people were scared to go to the facilities (2) Misinformation and misconception interfered with access due to COVID-19	Health worker hesitancy and misinformation; fear of unknown disease; lack of PPE	Fear of infection reduced demand for FP services (4)		Fear of infection
**Disruption of services / difficulty with lockdown & travel restrictions**	Services never stopped; little effect; no particular problem Lockdown restricted access (2) Service reductions affected access (2) Transportation issues disrupted health care workers Elective (permanent methods) postponed Low attendance at health centers (2) Impact of COVID-19 mostly at national level Declined overall performance of the program	All services put on hold during lockdown (2), with restrictions greatest during partial lockdown (3) Mobility to health facilities and personnel was interfered with During intense lockdown, mainly pregnant women moved easily to health facilities, not routine FP services Lockdown exacerbated challenges related to access to contraception for almost 2 years Disruptions were temporary (3) Restrictions short lived - deliberate initiative to continue essential health service delivery Access to services disrupted; counseling affected; management of side effects and concerns (3) Discouraged discontinuation due to limited access	Difficulties with lockdown (2) Issues at first, then improved (2) Health facilities functioning ok; some local effects Some services hours reduced (2) Travel restrictions limited access Remote areas most affected Service providers out on quarantine Lack of clarity on what services are deemed essential Elective services suspended (2) E.g. IUD, BTL, NSV, etc.)	Services closed due to lockdown (7) Closings initially then progressive opening Services limited (6) Comprehensive health care units for adolescents suspended	Lockdowns made access to FP difficult; reduced demand Restrictions at the beginning; better now	Lack of health facilities Difficulties with lockdowns (2); clinics closed Travel restrictions limited access Restriction to abortion during COVID (2)
**Staff, facilities diverted to COVID-19**		Health workers focused on COVID-19 (2) Focus and attention directed to COVID-19 prevention, thus neglecting FP services	Service providers occupied with COVID services (3) Hospitals converted for COVID-19	Service provider shortages (2)	Staff diverted to COVID-19 (2)	
**Access to RH services & contraceptive methods affected**		Limited access to removal of IUD	Lack of some methods (implants and injections)	LARC access/removal (3)	Difficulty getting resupply methods	
**Shifts in services/ outreach affected**		Community outreach affected (4)	Clinics were ok; outreach was affected Moved to private services	Lack of transportation for motivators	Tried a digital health program (pilot) for clients during and after COVID-19	Shift in services; home delivery, remote consultations
**Interfered with logistics & Supplies, training & supervision; M&E**		Stockouts; logistics and supplies; shortage of supplies (7) Interstate lockdown interfered with supplies Interfered with training (3) and supportive supervision	Monitoring was challenging (2)	Difficulties with logistics and supplies (2) Interfered with M&E	Stockouts/import difficulties	
**Lack of attention to FP/SRH; financing reduced; diverted; partnerships affected**		Diversion of resources meant for FP to combat COVID-19 SRH services not prioritized during COVID-19 Health partners involvement in FP services Coordination meetings with all stakeholders were affected	Financing – government moved funding to COVID-19 (2)	Lack of government attention to FP		FP not a priority in the country, even before COVID-19 (2)

* **Summary includes analysis of 178 responses from 63 countries (15 responses from 7 countries in EECA; 27 responses from 12 countries in Asia; 32 responses from 9 countries in LAC, 12 comments from 5 countries in MENA; 41 comments from 13 countries in SSA-F; and 51 responses from 17 countries in SSA-A). No comments were received from 7 countries. Numbers in parenthesis indicate multiple respondents gave the same/similar responses.**

A respondent from Morocco reaffirmed the diversion of providers, saying,

“Access to FP services has been affected due to the mobilization of FP health professionals in the context of the COVID 19 pandemic” (Morocco)

A respondent from Bangladesh explained,

“Long national lockdown had a role in receiving services from facilities. This created challenges in travel and provider contact mostly. Discontinuation of advocacy and counseling made disruption of services and increased the threat of unwanted pregnancy” (Bangladesh)

Also in Asia, a respondent from Pakistan reflected,

“Lock downs and smart lock downs have had its toll on both the providers and the users besides interrupted supply chain” (Pakistan)

In line with findings shown in Table 1, the comments mostly indicated more issues with LAPM than with STM. A respondent in Eswatini noted that,

“Procurement of family planning commodities was greatly affected which led to serious shortages of all methods, due to international lockdowns which affected the supply chain” (Eswatini)

Another respondent from Eswatini added that the shortages especially affected rural areas.

The comments showed varying views, even within regions and countries, on how severe and how long-lasting the effects of COVID-19 were and the interactions of COVID-19 with other underlying issues affecting the family planning program. For example, one respondent from Uganda said,

“The COVID-19 impact on FP services was most severe in April-May 2020 but the program recovered from June 2020 onwards quite well, due to a deliberate initiative to continue essential health service delivery” (Uganda)

In contrast, another respondent from Uganda described the situation differently, saying,

“Lock down exacerbated the challenges related to access to contraception in Uganda for almost 2 years” (Uganda)

A respondent from Burkina Faso noted the effect of COVID-19 on partnerships that were implementing programming, explaining that the pandemic interacted with other stressors to affect family planning:

“With COVID, many partnerships have been suspended, jeopardizing the progress of community interventions, especially in the context of insecurity with restrictions on movement in certain localities and the massive internal displacement of populations” (Burkina Faso)

A respondent from El Salvador explained that family planning got lost with COVID-19, saying,

“The priority of the government has been almost absolutely to care for COVID and a new maternal and childcare program which has been much publicized, but that leaves the family planning program abandoned” (El Salvador)

A respondent from Vietnam reflected on issues of equity, noting that,

“Those most affected by the COVID pandemic are those living in isolation and lockdown…through… the end of the third quarter of 2021 due to the Government’s Zero COVID strategy….Nearly half of the provinces live in a state of social distancing. The supply of essential goods is greatly affected because the list of essential goods is not clear…. in mountainous areas, economic conditions are difficult, the point of providing contraceptives is also limited compared to urban areas, they have fewer opportunities and choices” (Vietnam)

Not all respondents thought COVID-19 had adversely affected contraceptive use, although they did say that the family planning program had adapted to the pandemic conditions. A respondent from Tajikistan said,

“There was no decrease in contraceptive use during the Corona virus pandemic. More work was possibly done on the side of the healthcare professionals such as home delivery to patients who had COVID, etc., as well as provision of consultation remotely.”

Likewise, a respondent from Guinea explained that the effect was short-lived, saying,

“The COVID - 19 pandemic has hardly affected the use of FP services in Guinea. A 10% drop in use was recorded during the first month after the outbreak of the pandemic and immediately after, with the measures taken to maintain essential services including FP, usage gradually increased continuously and stabilized.”

Comments across the regions paint a picture of fear of COVID-19, along with lockdowns of varying durations, keeping people from accessing services, along with providers being diverted to COVID-19 services or being out sick themselves with COVID-19.

## Discussion

These findings from the 2021 NCIFP and its Supplement on COVID-19 provide a broad view from 70 counties across six regions of the resilience of family planning programs during COVID-19 into the second year of the pandemic. Our analysis shows that programs in all regions were affected by the COVID-19 pandemic. The magnitude of the effects varies by region and country, and by component of resilience. Comments across the regions paint a picture of fear of infection, with lockdowns of varying durations, travel restrictions keeping people from accessing services, and providers being diverted to COVID-19 services or being out sick themselves with COVID-19. While comments mostly implied that the effects were strongest early in the pandemic, that view was not uniform, with some respondents noting long periods of lockdown, for example, related to zero-COVID-19 policies. Similarly, respondents across four countries in a study by Brunie *et al.* (2022) reported that temporary service closures, product shortages, and fear of COVID-19 infections affected their access to and use of contraception. The comments in our study show differing views on the effects of COVID-19 for the same country, with respondents in one country reporting ‘no effect’ to a ‘lasting effect’. These comments illustrate the uncertainty based on the unknown with COVID-19: how long it would last; how severely it would affect different countries; and what services, including family planning and reproductive health, would be deemed essential and thus maintained throughout the pandemic.

While the average score for resilience, at 47 out of 100, implies middling levels of resilience, further analysis suggests that, for the most part, family planning programs were able to maintain government commitment and provide access to contraception despite facing challenges to financing; advocacy and community mobilization efforts; supply of contraceptives; routine data recording and reporting; and restrictions to movement/transport that interfered with the population’s access to STM and LAPM. Programs in Francophone Sub-Saharan Africa appeared to have been the most resilient, while programs in Latin America and the Caribbean appeared to have been most severely affected by, and thus least resilient to, COVID-19. Arsenault *et al.* (2022) found large declines in family planning services in the two LAC countries included in their study. The differences between LAC and SSAF-F may be due to the relatively high use of contraception in LAC compared to SSAF-F, with less government focus on family planning programming in countries in LAC than in SSAF-F, which was possibly shored up by more donor funding. Across the regions, COVID-19 had the largest negative impact on advocacy and community mobilization efforts. This could have been due partly to lockdowns that restricted external movements, shifting attention to COVID-19 related behaviors including wearing masks, keeping distance, and washing hands, among others.

This paper finds that despite ‘interference’ in many components of family planning programming, with some exceptions, respondents said that the COVID-19 pandemic generally did not diminish government commitment to family planning. Overall, programs remained resilient in providing access to services. Strong global attention to commodities and related supplies likely bolstered countries’ ability to provide access to services ( Weinberger *et al.*, 2023). Some countries that did not score highly on resilience reported indifferent commitment from governments even before COVID-19. This paper shows that a strong enabling environment for family planning, which the NCIFP is designed to measure, was positively correlated with continued government commitment and access to contraceptive methods during COVID-19, despite noted disruptions to services. This finding is supported by evidence from 15 countries that contraceptive use mostly increased over the years of the pandemic ( Bietsch *et al.*, 2022), and from analysis of successive waves of PMA ( https://www.pmadata.org/) data from four countries in sub-Saharan Africa ( Wood *et al.*, 2021).

### Limitations

This paper has limitations in that the findings from the countries are based on self-reports from respondents on their perceptions of the effects of COVID-19 on the family planning program. Still, the findings represent expert opinion from respondents in each country who were familiar with the family planning program and were in a unique position to observe program features and effects. The questions on COVID-19 were added as a Supplement to take advantage of the timing of the 2021 NCIFP, which limited the number of questions that could be added. That is, the respondents answered the questions on COVID-19 in the context of earlier items on the enabling environment for family planning. While a strength of the data is that they were collected in late 2021 and thus provide a broad perspective over the period of the pandemic from 70 countries across six regions, they do not capture any periodicity of the effects (e.g., effects of lockdowns with easing over time). The qualitative findings do indicate a gradation of effect, with the most intensive effects early in the pandemic. Further in-depth studies to examine the challenges faced by the programs and how they were overcome, or not, would add to our understanding of the resilience of family planning programs.

## Conclusion

The 2021 round of the NCIFP provides a unique view of the effects of the COVID-19 pandemic on family planning programming in 70 countries across six regions over two years of the pandemic. The questions added as a ‘COVID-19 Supplement’ to the standard questions in the NCIFP, an ongoing survey on the enabling environment for family planning, gauged both interference with various components of family planning and the extent to which governments maintained their program commitments and public access to services. Together, the questions measured the resilience of family planning programs in the face of COVID-19. The findings in this paper are instructive for family planning programming moving forward: it will face challenges and ‘interference’ when unanticipated shocks like COVID-19 occur, and strong programs will be best prepared to exhibit resilience during unexpected times.

## Data Availability

Zenodo: 2021 National Composite Index for Family Planning (NCIFP): Data File and Questionnaire. https://doi.org/10.5281/zenodo.8264220 ( Rosenberg *et al.*, 2022) This project contains the following underlying data: 2021 NCIFP Data File.xlsx (The data file includes the final, cleaned data for the 2021 round of the NCIFP, as well as a codebook identifying the variable names with their corresponding questions. Underlying country data are available from the authors upon reasonable request.) 2021 Questionnaire_English.pdf (The questionnaire is the full questionnaire for the 2021 round of the NCIFP, in English). Data are available under the terms of the Creative Commons Attribution 4.0 International.

## Footnotes

^1^ NCIFP data, reports, and country briefs are available at: http://www.track20.org/pages/data_analysis/policy/NCIFP.php
