# Supplementary material for: How resilient were family planning programs during the COVID-19 pandemic?  Evidence from 70 countries
Source: Gates Open Res. 2024 Mar 25;7:121. Originally published 2023 Sep 28. [Version 2] doi: 10.12688/gatesopenres.14856.2 (PMC11111842; doi:10.12688/gatesopenres.14856.2)
Supplement: Peer review history

# Reviewer report: Reviewer response for version 2

**DOI:** 10.21956/gatesopenres.16858.r36839

**Published:** 2024-07-23

**Authors:** Dr Kate Cheney

**Affiliations:** 1 The Sydney School of Nursing and Midwifery, The University of Sydney, Sydney, Australia

Thank you for submitting your manuscript; How resilient were family planning programs during the COVID-19 pandemic?  Evidence from 70 countries

 What an interesting question, from a planning perspective.

**Abstract:**

 - “…fears of severe disruptions to family planning (FP) and …” *– fear by whom?* *You might not have enough words to include a response but it raised the question for this reader.*

 - Can the  National Composite Index for Family Planning (NCIFP) DATA be briefly introduced as understanding who you are asking to respond is important to understand. 

**Background**

 Should you write out WHO in full first?

 What do you mean by 102’ states’ that responded, do you mean countries? You use both words

 “ Temporary service closures, product shortages, and fear of COVID-19 infections were reported to affect access and use. Using multiple rounds of Performance Monitoring and Action (PMA) data from Kenya, Burkina Faso, Lagos State Nigeria and Kinshasa, DRC, Wood *et al.* (2021) [Ref-1] reported some increases in women’s need for contraception" - *why was the need increasing  because of COVID? *Do you mean there was an unmet need? Or maybe that more women were asking for contraception for some reason related to the situation.

 ‘Most studies that have asked…’ ….can you provide some references?

 Again, what is the National Composite Index for Family Planning – a brief earlier introduction might be useful but I see you move to explain it in methods.

**Methods**

 Does the representation include Oceania?

 Just a comment , Asia has included some quite different countries grouped as one area.

**Discussion**

 This paragraph is key “This paper finds that despite ‘interference’ in many components of family planning programming, with some exceptions, respondents said that the COVID-19 pandemic generally did not diminish government commitment to family planning.”

**Limitations**

 “This paper has limitations in that the findings from the countries are based on self-reports from respondents on their perceptions of the effects of COVID-19 on the family planning program”

 SO , could it be that the NCIFP uses who they consider experts in FP  programming to answer this survey so those ‘experts’ may have political biases about governments who were in power over this time? They may have had their own COVID response biases? - just a thought and not a criticism or additional comment needed.

Is the work clearly and accurately presented and does it cite the current literature?

Yes

If applicable, is the statistical analysis and its interpretation appropriate?

I cannot comment. A qualified statistician is required.

Are all the source data underlying the results available to ensure full reproducibility?

Yes

Is the study design appropriate and is the work technically sound?

Yes

Are the conclusions drawn adequately supported by the results?

Yes

Are sufficient details of methods and analysis provided to allow replication by others?

Yes

Reviewer Expertise:

I am an academic midwife and nurse working around the area of contraception, with an interest in postpartum contraception. I was awarded a PhD around pregnancy planning and contraception, and have published in peer review journals and am a current peer reviewer. My current project is in a project about postpartum contraception and training in PNG, Samoa, Kiribati and Fiji.

I confirm that I have read this submission and believe that I have an appropriate level of expertise to confirm that it is of an acceptable scientific standard.

## References

[1] : Need for and use of contraception by women before and during COVID-19 in four sub-Saharan African geographies: results from population-based national or regional cohort surveys. *Lancet Glob Health* .2021;9(6) : 10.1016/S2214-109X(21)00105-4 e793-e801 10.1016/S2214-109X(21)00105-4 34019835 PMC8149322

# Reviewer report: Reviewer response for version 2

**DOI:** 10.21956/gatesopenres.16858.r36260

**Published:** 2024-07-23

**Authors:** Nadia Diamond-Smith

**Affiliations:** 1 Institute for Global Health Sciences, University of California San Francisco, San Francisco, California, USA

The authors responded to some of the comments from my prior review.

Is the work clearly and accurately presented and does it cite the current literature?

Yes

If applicable, is the statistical analysis and its interpretation appropriate?

Partly

Are all the source data underlying the results available to ensure full reproducibility?

Yes

Is the study design appropriate and is the work technically sound?

Yes

Are the conclusions drawn adequately supported by the results?

Yes

Are sufficient details of methods and analysis provided to allow replication by others?

Yes

Reviewer Expertise:

reproductive and maternal health, demography

I confirm that I have read this submission and believe that I have an appropriate level of expertise to confirm that it is of an acceptable scientific standard.

# Reviewer report: Reviewer response for version 2

**DOI:** 10.21956/gatesopenres.16858.r36259

**Published:** 2024-04-01

**Authors:** Philip Anglewicz

**Affiliations:** 1 Department of Population, Family and Reproductive Health, Johns Hopkins Bloomberg School of Public Health,, Baltimore,, MD,, USA

The authors have adequately addressed my previous comments.

Is the work clearly and accurately presented and does it cite the current literature?

Yes

If applicable, is the statistical analysis and its interpretation appropriate?

Yes

Are all the source data underlying the results available to ensure full reproducibility?

Yes

Is the study design appropriate and is the work technically sound?

Partly

Are the conclusions drawn adequately supported by the results?

Yes

Are sufficient details of methods and analysis provided to allow replication by others?

Yes

Reviewer Expertise:

Demography, family planning, survey methods.

I confirm that I have read this submission and believe that I have an appropriate level of expertise to confirm that it is of an acceptable scientific standard.

# Reviewer report: Reviewer response for version 1

**DOI:** 10.21956/gatesopenres.16184.r35589

**Published:** 2024-05-22

**Authors:** Luis Bahamondes, M. Valeria Bahamondes

**Affiliations:** 1 Department of Obstetrics and Gynecology, University of Campinas Faculty of Medical Sciences, Campinas,, SP, Brazil; 2 Independent consultant, Austin, tx, USA

Thank you for the opportunity to review the manuscript: How resilient were family planning programs during the COVID-19 pandemic?  Evidence from 70 countries.

 Background: “At the beginning of the COVID-19…” Please rephrased as: At the beginning of the SARS-CoV-2 (COVID-19)… Also at introduction for the first time.

 What means “M&E”? Please don´t use acronyms without explanation.

 There are no doubts that the COVID-19 pandemic affected in major or minor severity the FP services and access of women and men not only for services but also for contraceptive methods and it was affected different countries.

 One of the main limitations is that in LAC the authors did not evaluate large countries like Brazil, Mexico and Argentina with severe lockdown and negationist perspectives about COVID-19 from the governments of the two largest countries.

 I suggest visiting the paper (Charles et al, 2022) ^1^ About the sales of contraceptive methods in Brazil.

 In conclusion, the manuscript is well writing, and the study was well conducted.

Is the work clearly and accurately presented and does it cite the current literature?

Yes

If applicable, is the statistical analysis and its interpretation appropriate?

Yes

Are all the source data underlying the results available to ensure full reproducibility?

Yes

Is the study design appropriate and is the work technically sound?

Yes

Are the conclusions drawn adequately supported by the results?

Yes

Are sufficient details of methods and analysis provided to allow replication by others?

Yes

Reviewer Expertise:

Contraception

We confirm that we have read this submission and believe that we have an appropriate level of expertise to confirm that it is of an acceptable scientific standard.

## References

[1] : Comparison of contraceptive sales before and during the COVID-19 pandemic in Brazil. *Eur J Contracept Reprod Health Care* .2022;27(2) : 10.1080/13625187.2022.2027364 115-120 10.1080/13625187.2022.2027364 35156489

# Reviewer report: Reviewer response for version 1

**DOI:** 10.21956/gatesopenres.16184.r35588

**Published:** 2024-03-04

**Authors:** Philip Anglewicz

**Affiliations:** 1 Department of Population, Family and Reproductive Health, Johns Hopkins Bloomberg School of Public Health,, Baltimore,, MD,, USA

This study addresses a worthwhile point, that not much is known about exactly what aspects of FP programs may have been disrupted by COVID, and how this varies across countries.  This research uses data from key informants in 70 countries to estimate the impact of COVID, compare across and within regions, and provide more details on the impact.  

 Some strengths of this research include the consistent categorization of COVID-19 impacts, which permits a comparison across countries; the ability to identify what feature of the program was impacted; and the accompanying open-ended responses that provide more details.  So overall, this provides a high-level overview of COVID-19’s impact across settings, which has not been done to date.

 That said, a limitation of the approach is that it doesn’t measure the impact of COVID-19 directly, by asking women about their experience with accessing services, for example, but instead asks key informants who know the FP programs in their geography.  The disadvantage of this is that the key informants could be wrong, or at least inaccurate (in some or all sections)- just as many experts were in predicting the impact of COVID-19 at the beginning of the pandemic.  Was there any way to assess the validity of the responses?  Similarly, the NCIFP might be vulnerable to response bias, in which areas that were more affected by COVID-19 were less likely to respond.  Overall, this research provides valuable insight overall, but might benefit from a bit more information about the survey methods.

Is the work clearly and accurately presented and does it cite the current literature?

Yes

If applicable, is the statistical analysis and its interpretation appropriate?

Yes

Are all the source data underlying the results available to ensure full reproducibility?

Yes

Is the study design appropriate and is the work technically sound?

Partly

Are the conclusions drawn adequately supported by the results?

Yes

Are sufficient details of methods and analysis provided to allow replication by others?

Yes

Reviewer Expertise:

Demography, family planning, survey methods.

I confirm that I have read this submission and believe that I have an appropriate level of expertise to confirm that it is of an acceptable scientific standard, however I have significant reservations, as outlined above.

# Response

**Published:** 2024-03-20

**Authors:** Karen Hardee

**Affiliations:** Hardee Associates, USA

You raise a valid point that a limitation of our study is that it does not directly measure how women were actually impacted by the pandemic.   We have added a point in the methodology section on this, and have added text in the discussion about the need for further analysis, where data are available, linking NCIFP findings with data on use of family planning during the pandemic:  “While the NCIFP does not measure how women were actually impacted by the pandemic with regards to contraceptive access and use, NCIFP country managers (selected because of their known familiarity of the FP environment in their own countries) were instructed to ensure inclusion of FP advocates, gender-oriented NGOs, women’s  groups working on FP/RH—who are likely to get feedback from constituents about problems accessing FP during the pandemic.  We have included qualitative comments from respondents about key barriers in each country.

# Reviewer report: Reviewer response for version 1

**DOI:** 10.21956/gatesopenres.16184.r35241

**Published:** 2023-11-14

**Authors:** Nadia Diamond-Smith

**Affiliations:** 1 Institute for Global Health Sciences, University of California San Francisco, San Francisco, California, USA

Thank you for the opportunity to read this paper, entitled "How resilient were family planning programs during the COVID-19 pandemic?  Evidence from 70 countries". This paper presents data from surveys of experts in 70 countries about how COVID had impacted various components of FP and also the overall gov commitment to FP. Overall the paper is clearly written and has an interesting finding that there actually wasn't as huge of a disruption as many had feared. This paper does not involve very complicated analysis, but I think that the findings will be interesting to program and policy makers if the authors are able to better delve into more about why they think that some countries had more resiliency than others, there is a little in there, but I think this really needs to be fleshed out. The differences are the interesting part and help us know what to do to improve things. Saying that stronger programs with more commitment did better doesn't really help us that much and we probably would have known without this study. More detailed comments below. 

**Methods:**

- A little more detail about how respondents are selected would be helpful, is it word of mouth/connections? 
- Is it the same people who have answered across all years?
- The methods make it seem like you will be presenting longitudinal data, but I dont think that you actually do at all, so you might not want to focus on that as much. 

**Results/Discussion:**

- One thing that I kept struggling with is that these countries have different baseline levels of use of FP. I see you discuss this some in the discussion, but I think that you could discuss this even more and possibly do some analysis in the results of how baseline FP use is associated with resilience.
- Same could be said for donor $, I kept thinking about how countries that rely a lot on donor money probably would have different levels of resiliency, and again, you briefly mention this in the discussion, but is there a way to graph resiliency and donor $ % or something? 
- Also relatedly, the distribution of where the funding comes from seems important to discuss and how differences between countries might explain your findings. I think you are only measuring gov commitment and resilience, but some of these countries must get more or less $ from gov vs donors? 
- The r2 is pretty weak, the way it is presented/worded makes it sound stronger than it is.
- Hard to follow the acronyms ("with LAPM than with STM"), can you just write out? 
- I'm not sure the write in findings are that insightful (or at least not all of them) - you could potentially shorten this section. 
- I think more of the analysis suggested above could help us really situate these findings in the broader context and help us know how to prepare for the next disruption (what really works to help resiliency?) 

Is the work clearly and accurately presented and does it cite the current literature?

Yes

If applicable, is the statistical analysis and its interpretation appropriate?

Partly

Are all the source data underlying the results available to ensure full reproducibility?

Yes

Is the study design appropriate and is the work technically sound?

Yes

Are the conclusions drawn adequately supported by the results?

Yes

Are sufficient details of methods and analysis provided to allow replication by others?

Yes

Reviewer Expertise:

reproductive and maternal health, demography

I confirm that I have read this submission and believe that I have an appropriate level of expertise to confirm that it is of an acceptable scientific standard, however I have significant reservations, as outlined above.

# Response

**Published:** 2024-03-20

**Authors:** Karen Hardee

**Affiliations:** Hardee Associates, USA

Thank you for your comments on the paper.  We have incorporated responses as follows. 

- We have added more detail about how the respondents were identified for the survey, including the criteria for inclusion, and ensuring a range of perspectives on each country program, namely that respondents have at least 5 years’ experience with the FP program, and ensuring at least two respondents from each of the listed groups.
- We have added a sentence to explain that the same respondents were identified for the 2021 round of the NCIFP, but their inclusion was not always possible given turnover in relevant positions. 
- We have revised the methods section to clarify that we are not presenting longitudinal data. The COVID-19 questions were only asked in the 2021 round and the paper focuses only on that round. 
- It is true that the countries have different baseline levels of contraceptive use.  We have added to the limitations:  Characteristics of the family planning program before the onset of COVID-19 may have impacted resilience of the program including the strength of the health system, presence of donor support and funding, and socio-cultural factors. Beyond different levels of contraceptive use, method mix could also have an impact on resilience as countries with higher levels of long-acting method use may have had less interruption, since women wouldn’t have to come to the facility regularly for resupply.  Many factors may impact a country’s resiliency, and to varying degrees. The purpose of this analysis was to present the findings from the COVID-19 Supplement of the 2021 NCIFP and to provide a broad overview of the impacts of COVID-19 on national FP programs. Additional analyses exploring these and other factors would add to our understanding of FP program resilience and the characteristics that may lead to more or less reliance in the face of widespread disruptions. An in-depth analysis of these causes for individual countries is beyond the scope of this paper, however, we urge others to undertake further analysis. 
- We have added to purpose: Results are meant to serve as a starting point for policy and program managers to delve deeper into the root causes of resiliency in their respective countries and for researchers to gain a more nuanced picture of resiliency of family planning programing during the COVID-19 pandemic. 
- Financing is an important topic and warrants a separate paper that includes countries for which data on tracking of sources of funding is available. 
- We agree with the comment that one of the three r2 should not be characterized as strong.  We have modified the sentence:  The relationships for government commitment and access were strong, implying that maintaining government commitment to family planning and access to contraceptives during COVID-19 were both bolstered by a robust overall enabling environment for family planning.
- We have written out long acting and permanent methods and short term methods, with use of the acronyms in figures and tables. 
- We think the qualitative findings from regions add some depth to the findings and provide examples from various countries across the regions. We anticipate that readers will be interested in the insights from countries across the regions. 
- We agree that further investigation and analysis would add insight into resilience, we believe this sentence in the limitations section covers the point: “Further in-depth studies to examine the challenges faced by the programs and how they were overcome, or not, would add to our understanding of the resilience of family planning programs.”
